# Deep vein thrombosis resolution, recurrence and post-thrombotic syndrome: a prospective observational study protocol

**DOI:** 10.1186/s12878-016-0063-7

**Published:** 2016-09-15

**Authors:** M. Bonfield, F. Cramp, J. Pollock

**Affiliations:** 1 Vascular Studies Unit, University Hospitals Bristol NHS Foundation Trust, Bristol, UK; 2 University of the West of England, Bristol, UK

**Keywords:** Deep vein thrombosis, Post-thrombotic syndrome, Resolution, Recurrence, Anticoagulation

## Abstract

**Background:**

Reasons for the variation in response of deep vein thrombosis (DVT) to anticoagulation treatment are not known. Some patients develop complications such as post-thrombotic syndrome or recurrent DVT but others make a full recovery. The aim of the study is to identify the level of variation in response to anticoagulation treatment and provide more precise and quantitative disease characterisation in response to treatment.

**Methods:**

A prospective observational study using duplex ultrasound to examine changes in thrombus characterisation, evolution and resolution over a 2 year period in patients with a confirmed DVT. Logistic regression analysis will be used to seek associations between characteristics present at baseline and the outcomes of DVT resolution, recurrence and the development of post-thrombotic syndrome (PTS).

**Discussion:**

This research into the response to treatment of lower limb DVT and predictive factors for DVT resolution, recurrence and PTS could inform a more tailored approach to anticoagulation therapy for the future management of DVT.

UKCRN ID: 16016. Registered on 20 January 2014.

## Background

Deep vein thrombosis (DVT) is a blood clot or thrombus in one of the deep veins of the body. It is a common clinical condition which usually affects the deep veins of the lower limbs and abdomen. It is known to have a number of well-recognised, potentially life-threatening complications including pulmonary embolism as well as chronic long-term sequelae related to the lower limb itself, a condition known as post thrombotic syndrome (PTS) [1].

Research evidence to date suggests that there is variability in the response to treatment in terms of thrombosis resolution and residual venous function. Some thromboses may resolve before the course of anticoagulation treatment is completed and some may not be resolved upon completion of the treatment cycle. Some patients may recover fully from having had a DVT and others may be left with on-going problems associated with chronic venous hypertension. However, clinical ultrasound imaging is not routinely used on completion of the course of anticoagulation treatment to establish the status of the thrombosis or residual venous function. The length of treatment or the decision to stop or continue treatment is therefore not informed by either the degree of resolution of the DVT (i.e. the presence of residual thrombus) or the residual venous function. There is debate whether prolonged anticoagulation could benefit certain patient groups in terms of reducing the risk of recurrence and the development of PTS.

Not every patient that has a DVT will develop a recurrent DVT or PTS but there is a lack of research evidence to indicate which patients are at risk. Recurrent, ipsilateral DVT is the best established risk factor for PTS, increasing the risk as much as six-fold compared to patients without recurrence [2–5]. These studies however are 17–20 years old and are therefore based on old ultrasound technology. There is also no agreement regarding the impact of other factors such as DVT location or extent of thrombus resolution at treatment completion and the presence of residual thrombus, nor patient characteristics such as age, gender or body mass index on the risk of developing PTS.

New technological developments in the field of ultrasound allow for much improved visualisation of smaller vessel DVT and residual thrombus so there is justification for a new study in this field using the latest ultrasound technology. The research undertaken into the response to treatment of lower limb DVT and predictive factors for recurrence and PTS could inform a more tailored approach to anti-coagulation therapy for the future management of the presenting DVT and for preventing recurrence.

## Methods/Design

### Aims and objectives

a) Record thrombus morphology, evolution and resolution in a DVT patient population to identify the level of variation in response to anticoagulation treatment and provide more precise and quantitative disease characterisation in response to treatment.

b) To detect associations between characteristics present at baseline and outcomes identified during follow-up.

### Study design

A single centre prospective observational study.

### Study population

Patients referred to the rapid access Thrombosis Clinic at University Hospitals Bristol (UHBristol) for suspected DVT and subsequently diagnosed with a DVT on ultrasound. Patients are referred to the Thrombosis Clinic at UHBristol from both primary care and from within the hospital. The study population mainly comprises outpatients but patients diagnosed with a DVT through this service whilst an inpatient may be included in the study providing they meet the inclusion criteria.

### Inclusion criteria

Symptomatic adult patients aged between 18 and 85 years, diagnosed with a confirmed lower limb DVT on ultrasound. The diagnosis of lower limb DVT is made where partial or occlusive thrombus is present in any of the deep veins by either direct ultrasonic visualisation of intraluminal thrombus, lack of complete compressibility or absence of flow following distal compression.

### Exclusion criteria

Patients with mobility issues that prevent them returning for follow-up appointments or are unable to lie down or transfer to an examination couch, patients that are pregnant, those with active cancer and intra-venous drug users are excluded from the study. Patients were asked if they are participating in any other research when approached to consider participation in the study; if the other research study had the potential to confound this study, subjects were not recruited.

### Primary outcomes

The prospective observational study uses duplex ultrasound to examine changes in thrombus characterisation, evolution and resolution over time in patients with a confirmed DVT on ultrasound. The primary outcomes of the study are DVT resolution, recurrence and the development of PTS.

### Sample size calculation

The sample size calculation was based on the work of Peduzzi et al. [6] and reported by van Belle [7] as a guideline for the minimum number of subjects required to quantify regression coefficients with reasonable precision in an epidemiological study of this kind. The variables for examination will be identified from the research data as described previously so their precise number and their relationship with the primary outcome is not yet determined. Based on a calculation where p is the smallest of the proportions of negative or positive cases of PTS in the population and k the number of covariates, the minimum number of subjects to include can be calculated as: N = 10 k / p. Using research evidence that suggests that at least 20 % of patients with symptomatic DVT will develop PTS within 2 years despite ‘adequate’ treatment of DVT and assuming three covariates the minimum number of patients required would be 150.

### Study methods

To provide denominator data for the study, records have been kept of all patients that were referred to the Thrombosis Clinic service, UHBristol for ultrasound assessment for lower limb DVT during the recruitment period, including those with a negative diagnosis. Details have been kept of the recruitment process including the reasons for exclusion and where provided, reasons for patients’ refusal to participate.

On ultrasound diagnosis of a lower limb DVT, each potential participant was assessed for eligibility and, if appropriate, offered an invitation letter and patient information leaflet. If interested in principle, the patient was asked to provide a contact telephone number and convenient contact time. The chief investigator would then contact the potential participant by telephone after 24–48 h to ask them if they wished to participate in the study and to answer any questions they may have. If they agreed, a first follow-up appointment was scheduled for 7 days post diagnosis. Written consent to participate in the study was obtained from each participant at the first follow up visit by the chief investigator in accordance with Good Clinical Practice. Each participant will remain in the study for 2 years post diagnosis.

Complete diagnostic ultrasound assessment of the proximal and distal lower limb veins using a combination of colour and spectral Doppler and compression ultrasound is undertaken on initial presentation. The diagnostic ultrasound is undertaken by Clinical Vascular Scientists from the Vascular Studies Unit, UHBristol who are trained locally under the standards of the National School of Healthcare Science NHS Scientist Training Program. The unit performs between 2000 and 2400 diagnostic ultrasound assessments for DVT annually and has a detection rate of 20–24 %. The following details are recorded: thrombosis location and extent, if the thrombosis is occlusive or partially occlusive and if adherence to the vessel wall is complete or partial. Any relevant clinical history is recorded at initial presentation including the nature and duration of any clinical symptoms.

The presence of any pre-existing medical conditions is recorded at each participant’s first follow-up visit, as well as the baseline details for gender, age and body mass index. Details are recorded of all prescription medications currently being taken by each participant and of any non-prescription medications/vitamins/supplements being taken regularly. Any changes in medical history, prescription medicines and body mass index are recorded at each study visit.

The follow-up schedule comprises six visits for those participants with a first episode of DVT in the limb concerned at the following intervals: 1 week, 1 month, 3 months, 6 months, 1 year and 2 years. Participants with a history of previous ipsilateral DVT will undergo three follow up visits at 1 week, 6 months and 2 years.

At each visit participants undergo full compression ultrasound assessment of the complete lower limb venous tree from the level of the inguinal ligament to the ankle (common femoral vein, popliteal vein, peroneal veins, posterior tibial veins, anterior tibial veins, soleal veins, gastrocnemius veins, greater saphenous vein and short saphenous vein) to identify the presence or absence of residual or new DVT or superficial thrombophlebitis in each venous segment. The ultrasound assessment is undertaken by the chief investigator who is a Health and Care Professions Council (HCPC) registered Clinical Vascular Scientist and is professionally accredited by the Society for Vascular Technology of Great Britain and Ireland (SVTGBI). The presence of thrombus is recorded as occlusive or non-occlusive and details of recanalization patterns are recorded in each segment. If the original diagnostic scan identifies the presence of thrombus in the iliac veins then the abdominal venous system is also scanned in full at each follow-up visit. In all cases, if flow in the common femoral vein on spectral Doppler ultrasound is not phasic with respiration, a full assessment of the external iliac vein, common iliac vein and inferior vena cava is undertaken using colour and spectral Doppler ultrasound.

Fifteen venous segments are assigned a thrombus score at each visit: inferior vena cava, common iliac vein, external iliac vein, common femoral vein, profunda femoris vein, proximal femoral vein, distal femoral vein, popliteal vein, peroneal veins, posterior tibial veins, anterior tibial veins, soleal veins, gastrocnemius veins, greater saphenous vein and short saphenous vein.

The thrombus load score is adapted from the updated reporting standards in venous disease from the Ad Hoc Committee on Reporting Standards of the Joint Council of the Society for Vascular Surgery and the North American Chapter of the International Society for Cardiovascular Surgery [8] and a modified version of Haenan’s clot score as used by van Rij et al. [9].

Each venous segment is scored as follows:

- 0 = patent
- 1 = subsegmental, non-occlusive thrombus
- 2 = subsegmental, occlusive thrombus
- 3 = occlusive thrombus throughout segment

A duplex ultrasound assessment for the competency of the deep and superficial veins is undertaken by the chief investigator at each visit. Pulsed and colour Doppler is utilised to assess flow characteristics within the veins including phasicity, spontaneity and direction of flow. Flow characteristics are assessed in a longitudinal scan plane. The patient are examined standing or at a minimum of 30° degree angle on a tilting table in order to assess the competency of the venous valves against gravity. Manual distal compression is applied to the calf to augment the flow and to assess for reflux. Venous incompetence is defined as a reflux time >0.5 s [10].

At each visit, a detailed clinical assessment is made of the leg in which the thrombosis is/was present and the patients’ symptoms, including pain, aching, heaviness, itching, cramp and paraesthesia are recorded using an interviewer-administered questionnaire. The Villalta Scale [11] will be used to quantify the symptoms of the burden of venous disease. The presence of oedema, teleangiectasias, reticular veins, varicose veins, hyperpigmentation, eczema, lipodermatosclerosis and ulceration are recorded according to the Clinical, Etiological, Anatomical and Pathophysiological (CEAP) classification system for classifying venous disease severity.

Details of the anti-coagulation treatment received by each patient in the study are collected as it is recognised that there are several different anti-coagulation therapies available and that variation in anti-coagulation regimes can occur depending on patient pathway, patient profile, patient choice and primary care follow-up. Adequacy of anticoagulation therapy is monitored through access to the participants’ medical records where relevant (vitamin K antagonists where international normalized ratio (INR) is monitored) and compliance with taking new oral anticoagulants (NOACs) is assessed by direct questioning at each visit. Participants’ compliance with compression stocking use is also recorded at each visit as ‘no use’, ‘occasional use’ or ‘routine use all day, every day’.

Any participant identified as having a recurrent DVT or an existing DVT that has extended at any point during follow-up is immediately reviewed by the rapid access thrombosis clinic. A recurrent DVT is diagnosed as the presence of a new thrombus either in the ipsilateral or contralateral leg or the presence of new thrombus on old thrombus in the same venous section as the original thrombosis, all of which are confirmed on Duplex ultrasound.

### Data analysis

International Business Machines Corporation Statistical Product and Service Solutions (IBM SPSS) Version 20 (IBM, Armonk, New York, USA) will be used for numerical operations. Initial analysis will involve running descriptive statistics. Individual and multi-sector vein thrombus presence and change will be analysed for patterns and categories of resolution and recurrence over the 48 month follow-up period. Frequencies will be performed for each predictor in addition to the sample size and age and sex distributions. Crude odds ratios and their confidence limits will be calculated to describe the strength of association between each independent variable and the primary outcome variables [12]. A chi-square test of independence will initially be performed to highlight the categorical variables with a significant relationship to the dependent variable for inclusion in regression modelling. Logistic regression analysis will be used to compute the adjusted regression coefficients and confidence limits of variables predictive of DVT recurrence and PTS.

The timing of any DVT recurrence and post thrombotic syndrome will be assessed using survival analytical methods such as Kaplan and Meier [13], with Cox regression employed to quantify covariate hazard rates.

The variance inflation factor will be used to identify any concerns that multicollinearity poses a threat to the regression analysis and to determine the individual importance of each predictor [14]. An analysis of the basic residual statistics of the model will be undertaken to identify cases exerting undue influence over the parameters of the model [14].

## Discussion

### Study status

Since February 2014, 195 participants were recruited to the study. The study closed to recruitment in January 2016 and the first participants completed 2 years of follow up in February 2016. Data collection will continue until January 2018 with results expected to be published in late 2018.

## Abbreviations

CEAP: Clinical, Etiological, Anatomical and Pathophysiological classification system
DVT: Deep vein thrombosis
IBM SPSS: International Business Machines Corporation Statistical Product and Service Solutions
INR: International normalised ratio
NHS: National Health Service
NOACs: New oral anticoagulants
PTS: Post-thrombotic syndrome
UHBristol: University Hospitals Bristol
